# High-Temperature Corrosion of Different Steels in Liquid Sn-Bi-Zn Heat Transfer Alloy

**DOI:** 10.3390/ma18092149

**Published:** 2025-05-07

**Authors:** Qingmeng Wang, Xiuli Wang, Xiaomin Cheng, Qianju Cheng, Yi Yang

**Affiliations:** 1 School of Mechatronics and Intelligent Manufacturing, Huanggang Normal University, Huanggang 438000, China; wangqingmeng@whut.edu.cn (Q.W.);; 2 Hubei Xinghe Electric Power New Materials Co., Ltd., Huanggang 438000, China; 3 School of Materials Science and Engineering, Wuhan University of Technology, Wuhan 430070, China

**Keywords:** Sn-Bi-Zn alloy, heat transfer fluids, high-temperature corrosion, diffusion, dissolution

## Abstract

In the fields of nuclear engineering and solar thermal utilization, low melting point alloys with excellent thermal conductivity and heat transfer performance have attracted extensive research as a new generation of heat transfer fluids, leading to many fundamental and important application issues. This study investigates the high-temperature corrosion behavior of Sn-50Bi-2Zn (wt.%) heat transfer alloy against 304 stainless steel (304), 310S heat-resistant steel (310S), and 20 carbon steel (20C) at 600 °C. Theoretical analysis, based on Fick’s diffusion law, and experimental measurements reveal significant differences in corrosion severity. After 473 h, 20 carbon steel exhibited the lowest corrosion layer thickness (0.07 mm), while 310S suffered the most severe corrosion (1.50 mm), exceeding 304SS (0.83 mm) by 81%. Diffusion coefficients derived from Sn penetration depths further quantified these trends: D310S = 2.51 × 10^−7^ mm^2^/s (6.8 × higher than 304: 3.7 × 10^−8^ mm^2^/s) and D20C = 2.87 × 10^−10^ mm^2^/s (128 × lower than 304SS). XRF analysis confirmed the dissolution of steel components into the molten alloy, with Fe, Cr, and Ni content increasing to 0.382 wt.%, 0.417 wt.%, and 0.694 wt.%, respectively, after 480 h. These results underscore the critical role of Ni content in accelerating Sn/Zn diffusion and pore formation, providing actionable insights for material selection in high-temperature heat transfer systems.

## 1. Introduction

In the field of energy, efficient heat transfer systems are crucial for improving energy utilization efficiency. The Sn-50Bi-2Zn (wt.%) alloy, with its low melting point (~138 °C for Sn-Bi eutectic), high thermal conductivity (~25 W/m·K), and excellent fluidity [1,2,3], is particularly suited for applications requiring efficient heat transfer under moderate to high temperatures. For example, it has great potential in the fields of solar thermal power generation systems [4,5] and computer chip heat dissipation [6,7]. Especially after the Fourth Generation International Forum [8,9], liquid metals were identified as heat transfer agents for nuclear reactors. Steel pipelines are widely used in high-temperature heat transfer systems in many industrial fields due to their high strength, good thermal stability, and relatively low cost. Common steel pipeline materials used in high-temperature environments include alloy steel pipes such as 15CrMo and 12Cr1MoV, as well as carbon steel pipes such as 10 #, 20 #, and 45 #, and stainless steel pipes such as 304 and 316 [10,11,12]. Different steel pipeline materials have their own unique performance characteristics, which make them suitable for different high-temperature heat transfer conditions.

However, when heat transfer alloys come into contact with these steel pipeline materials at high temperatures and undergo heat transfer processes, a problem that cannot be ignored arises, which is high-temperature corrosion [13,14,15,16]. Under high temperature conditions, the elements in the alloy may undergo complex chemical reactions with the components in the steel pipeline material, leading to the formation of corrosion products on the surface of the pipeline, thereby changing the surface properties and internal structure of the pipeline. This may not only reduce the heat transfer efficiency of the pipeline but also cause safety hazards such as pipeline leaks, ultimately affecting the reliable operation of the entire high-temperature heat transfer system [17,18].

Early research on corrosion mainly focused on the diffusion behavior of elements such as Sn, Bi, Fe, and Ni [19,20,21]. Meanwhile, S.K. Kang [22,23] calculated in detail the corrosion kinetics of Ni dissolved in liquid Sn at 270 °C, 350 °C, and 400 °C. Based on this, Thomas Emmerich [24] studied the high-temperature corrosion between Ni-based alloys and liquid Sn. However, the specific mechanism by which Bi and Sn inhibit the reaction of steel in liquid zinc has not been fully elucidated. In recent years, the corrosion direction of low melting point alloys has mainly focused on the behavior and mechanism of multi-component packaging materials. Zhang et al. [25] discussed the corrosion of liquid metals with steel and established a corrosion model. Zhang simulated the mass transfer process at the contact surface of liquid metals flowing on the steel surface. E. Yamaki [26] used 316L stainless steel as a packaging container to analyze its dissolution mechanism in Pb-Bi liquid alloy. Henryk Kania [27] found that the dissolution of steel in a zinc bath containing Bi and Sn follows a linear pattern. Bi and Sn can lower the dissolution constant and increase the activation energy, thereby inhibiting iron dissolution. Yi-Jia Li et al. [28] studied the effect of Sn addition (0–2 wt%) on the microstructure and corrosion behavior of Mg-2Zn-0.5Ca alloy. The results showed that the alloy had the lowest corrosion rate and the best comprehensive performance when 1 wt% Sn was added. However, the complex interaction between Sn and other elements was not deeply studied, and the experiment did not cover the corrosion behavior under a high-temperature environment.

This article aims to clarify the chemical reaction pathways, composition, and structure of corrosion products, and influencing factors of corrosion rate during the corrosion process through a combination of experimental such as materials preparation, corrosion testing, microstructural and elemental characterization and theoretical analysis such as Fick’s diffusion law, thermodynamic and kinetic interpretations. It analyzes the high-temperature corrosion behavior of Sn-Bi-Zn heat transfer alloys on different steel pipeline materials, providing a solid theoretical basis and practical guidance for material selection and operation maintenance of high-temperature heat transfer systems.

## 2. Materials and Methods

### 2.1. Materials and Preparation

Sn-50Bi-2Zn alloy refers to weight percentages (wt.%). The raw metals—Sn (tin), Bi (bismuth), and Zn (zinc)—were purchased from Aladdin Reagent Co., Ltd. (Shanghai, China), with a purity of 99% for all three metals. The designed alloy was melted in a low-power melting furnace at 350 °C. During the experiment, high-purity argon protection was introduced to prevent the compositional changes caused by the liquid surface oxidation of the alloy. The melted samples were poured into the iron mold with a size of φ30 × 100 mm.

### 2.2. Corrosion Experiment

The container materials used in the corrosion test are 304 stainless steel, 310S heat-resistant steel, and 20 carbon steel. Drill holes in three different types of steel plates, string them together with steel wires, and place them in a container. After the prepared Sn, Bi, and Zn samples are completely melted at 200 °C, they are poured into stainless steel pipes and air cooled. After the metal liquid solidifies and cools, the open part of the steel pipe is welded with steel plates to ensure that the steel plates are completely sealed with the metal liquid, numbered 1 #, 2 #, 3 #, 4 #, and 5 #. The stainless steel pipes containing steel sheets and molten metal were placed into a well furnace, maintaining the temperature inside at around 600 °C, and taking out sample pipes numbered 1 # to 5 # in sequence every 100 h.

### 2.3. Phase and Microstructure Experiments

The metallographic structure of the container material before and after corrosion was observed, and its metallographic structure was photographed using a metallographic microscope (OM, Leica DM6M, Leica Microsystems, Wetzlar, Germany). The microscopic morphology of the corrosion zone was observed by field emission scanning electron microscopy (FE-SEM, JEOL JSM-7500F, JEOL Ltd., Akishima, Tokyo, Japan), and then the elemental content of the corrosion zone was characterized by an energy dispersive spectrometer (EDS, INCAX-ACT, Oxford Instruments, Abingdon, UK). The corrosion thickness of the three steels was calculated. Sheets of different times are used for the corrosion kinetics process.

The surface of the steels after corrosion was investigated by X-ray diffraction (XRD, D8 ADVANCE, Bruker, Germany) equipped with a Cu Kα radiation source (wavelength: λ = 1.5406 Å), and the phase compositions of the alloys before and after corrosion were analyzed by X-ray fluorescence spectroscopy (XRF, AXIOS, PANalytical B.V, Almelo, Netherlands).

## 3. Results and Discussion

### 3.1. Metallographic Observation of Steel Sheets Before and After Corrosion

The experiment adopted the static uniform corrosion full-immersion test method. Steel sheets of 304 stainless steel, 310S heat-resistant steel, and 20 carbon steel were completely immersed in the liquid Sn-Bi-Zn metal at 600 °C in an air-isolated environment. The steel sheets were taken out at different time points to analyze the corrosion of the steel sheets by the molten metal.

**Figure 1:**
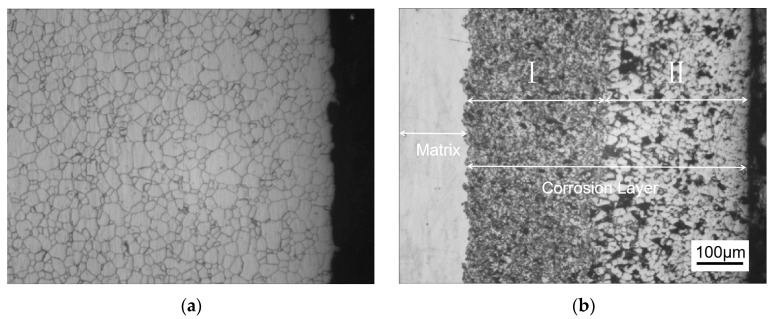
Metallographic diagrams of the cross-section of a 304 stainless steel sheet before and after corrosion. (**a**) Before corrosion, (**b**) After corrosion (I: Close to the stainless steel substrate. II: The transition zone).

Figure 1 shows the metallographic structure of the 304 stainless steel section before and after corrosion for 180 h. The results showed that the corrosion layer of 304 stainless steel had a clear boundary with the substrate, and part I was close to the stainless steel substrate, with smaller grain size and denser structure than the substrate; Part II is the transition zone between the edge of the steel sheet and Part I, with coarser grains and obvious granular shapes compared to Part I, and a large number of pores. In Part II, as the corrosion layer deepens, the size of the pores gradually increases, and the number decreases.

**Figure 2:**
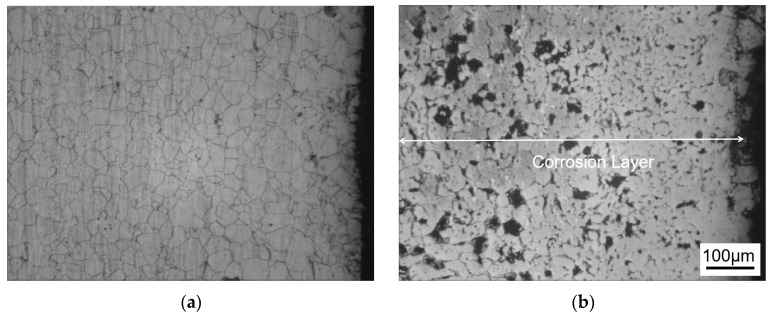
Metallographic diagrams of the cross-section of a 310S heat-resistant steel sheet before and after corrosion. (**a**) Before corrosion, (**b**) after corrosion.

Figure 2 shows the metallographic structure of 310S heat-resistant steel section before and after corrosion. The results showed that the corrosion of 310S heat-resistant steel was much more severe than that of 304 stainless steel. After 180 h of experiment, almost all of the steel plates used in the experiment were corroded, and the pores were distributed in various areas of the corrosion layer. There was no obvious interface distinction in the corrosion layer, but as the corrosion depth increased, the grain size became coarser, and the pores caused by corrosion became larger.

**Figure 3:**
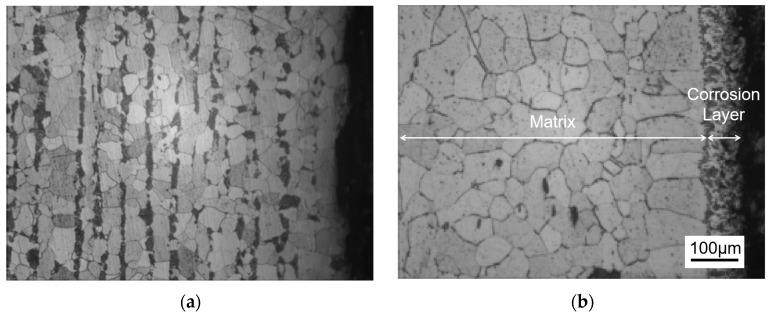
Metallographic diagrams of the cross-section of a 20 carbon steel sheet before and after corrosion. (**a**) Before corrosion, (**b**) after corrosion.

Figure 3 shows the metallographic structure of the cross-section of 20 carbon steel before and after corrosion for 180 h. The high-temperature resistant liquid Sn-Bi-Zn alloy of 20 carbon steel has good corrosion resistance, and the interface of the corrosion layer is obvious. It is composed of a very thin corrosion layer at the edge and the substrate. In the corrosion layer, Sn-Bi-Zn alloy mixes with the Fe substrate and corrodes from the outside to the inside, perpendicular to the interface. The grain size of the matrix increases compared to before corrosion, and the metallographic structure before corrosion consists of white ferrite and dark pearlite. After corrosion, the pearlite basically disappears, and black granular carbides are produced in the matrix area.

### 3.2. Micro-Area Corrosion Morphology and Element Distribution

#### 3.2.1. Element Distribution Results of 304 Stainless Steel After Corrosion

**Figure 4:**
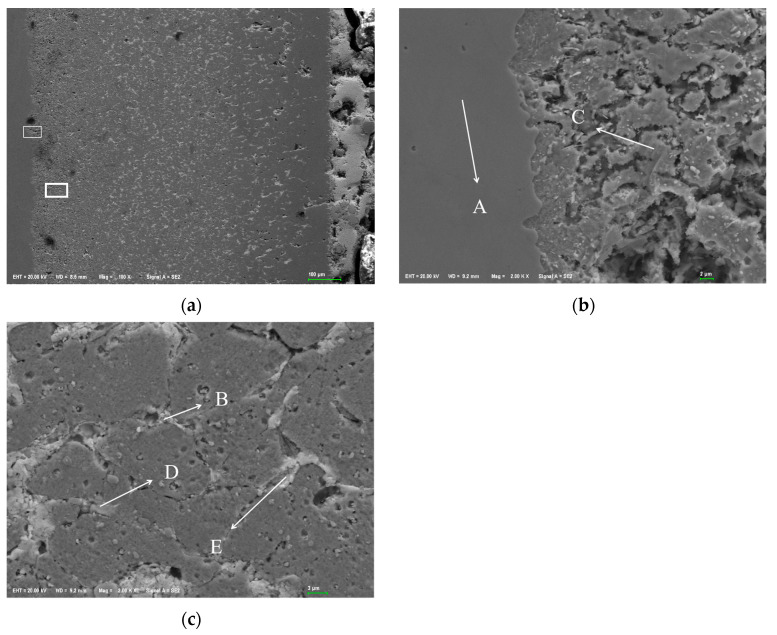
SEM morphology of corrosion layer on 304 stainless steel. (**a**) 100×; (**b**) 2000× (interface); (**c**) 2000× (corrosion layer).

Figure 4 shows the SEM corrosion morphology of a cross-section of 304 stainless steel. The dark gray area on the left is the Fe matrix, the light white area on the right is the Sn-Bi-Zn alloy, and the area filled with pores is the corrosion layer. From the figure, it can be seen that the contact area between the corrosion layer and the boundary is smooth and flat, with very few holes. As the corrosion deepens, the number of holes increases. The corroded area is filled with a large number of pores, and as the corrosion depth increases, the pore size decreases but becomes denser. Figure 4b,c is an enlarged partial view of the white box area in Figure 4a. It can be seen that the edges of the corroded layer grains are corroded into irregular sawtooth shapes by the alloy, and the pores between grains are more obvious.

Figure 4b shows the contact area between the corrosion layer and the substrate of 304 stainless steel, while Figure 4c shows the internal area of the corrosion layer. Table 1 lists the energy spectrum analysis results of marked points. During sample polishing, the diamond polishing agent introduced C and Si, which can be ignored in the analysis. At point A, the composition ratio of Fe, Cr, and Ni is similar to that of 304 stainless steel, indicating no infiltration of Sn, Bi, and Zn into the matrix. The boundary line in Figure 4b is the diffusion endpoint of these alloy elements. In the corrosion layer, Ni was undetected at points B, C, D, and E. B and C are incompletely corroded matrix areas mainly with Fe and Cr, where a small amount of Sn and Zn diffused, and the decrease in Cr and Ni increased Fe content. Points D and E fill the gaps between corroded grains. SEM shows D is like a whole, and E is a multi-layer structure. EDS reveals that D is mainly composed of Fe, Cr, and Sn with little Bi and Zn, while E is mainly Fe, Cr, and Bi with little Sn and Zn. Also, Sn and Zn atoms are more likely to diffuse into the grain than Bi atoms under high-temperature liquid corrosion of the ternary alloy.

**Figure 5:**
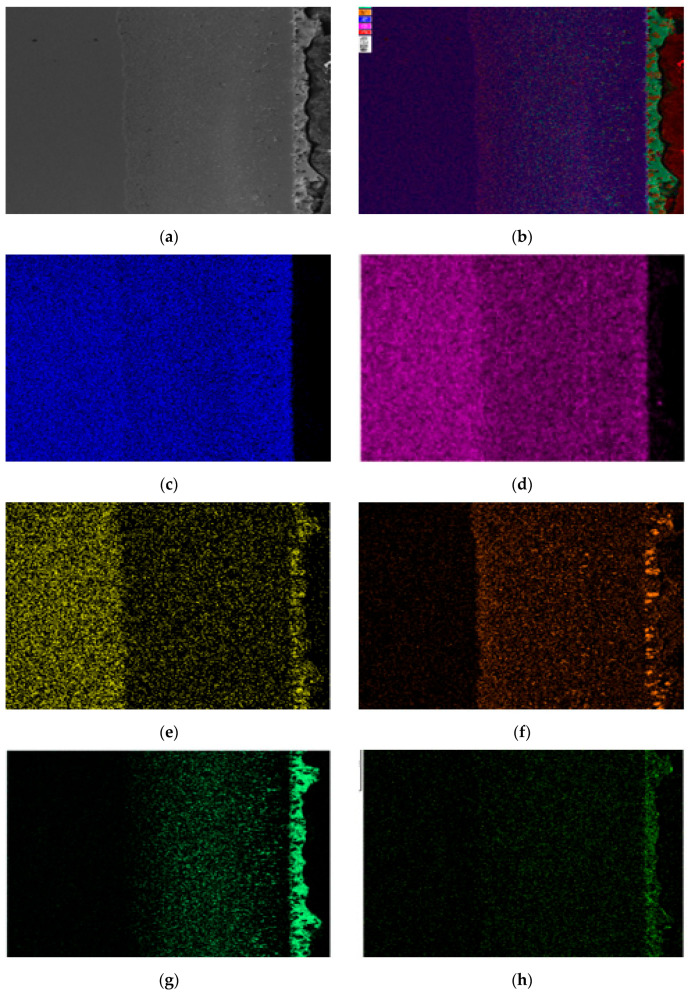
Element distribution of the cross-section of 304 stainless steel after corrosion. (**a**) Surface scanning area; (**b**) EDS layered image; (**c**) Fe; (**d**) Cr; (**e**) Ni; (**f**) Sn; (**g**) Bi; (**h**) Zn.

Figure 5 shows the EDS surface scan of the corroded section of 304 stainless steel. The results show that Sn, Bi, and Zn elements diffuse significantly in the stainless steel. Among them, Sn element is evenly distributed in the corrosion layer, while Zn element gradually decreases in concentration as the corrosion depth increases. Bi element is rarely distributed on the surface of the steel sheet and concentrated in the middle of the corrosion layer. There is a clear interface layer of Fe element in the energy spectrum, and the concentration change is not significant. The concentration of Cr and Ni elements in the corrosion layer is significantly lower than that in the stainless steel substrate, with the Cr element having a higher concentration in the corrosion layer and the Ni element having a significantly reduced concentration in the corrosion layer.

**Figure 6:**
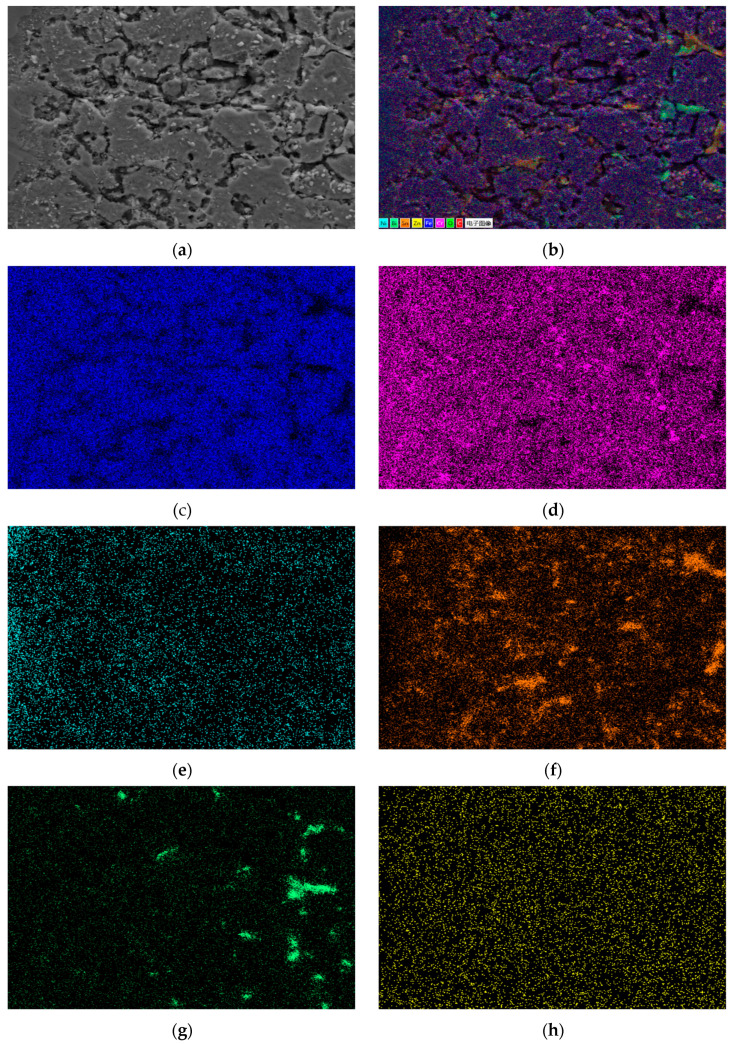
Micro-area EDS surface scanning results of the corrosion layer of 304 stainless steel. (**a**) Scanning area; (**b**) EDS layered image; (**c**) Fe; (**d**) Cr; (**e**) Ni; (**f**) Sn; (**g**) Bi; (**h**) Zn.

Figure 6 shows the surface scan of the corrosion micro zone of 304 stainless steel, which indicates the presence of Sn and Zn elements within the grains of the corrosion zone. The gaps in the corroded grain boundaries are filled with Sn and Bi elements, and Zn elements are evenly distributed but in small quantities. It can be seen that Sn and Bi elements are concentrated in different regions, with very little overlap. The distribution of the Cr element is uniform, overlapping with Sn and Bi elements in the gaps, and there is an enrichment phenomenon in some gaps. The concentration of the Ni element is low in the micro-EDS spectrum, indicating that the Ni element is severely lost during the corrosion process.

#### 3.2.2. Element Distribution Results of 310S Heat-Resistant Steel After Corrosion

**Figure 7:**
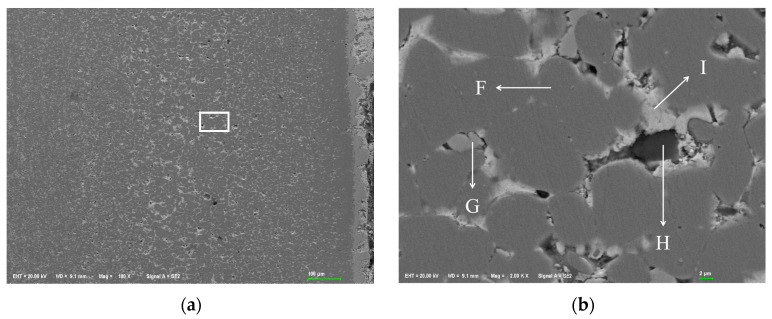
SEM morphology of corrosion layer on 310S heat-resistant steel. (**a**) 100×; (**b**) 2000×.

Figure 7a shows the SEM corrosion morphology of the 310S heat-resistant steel section. It can be seen that the steel interface has been completely corroded. The dark area on the left is the Fe-based corrosion layer, the middle boundary is the surface of the steel sheet, and the gray white layer on the right is the Sn-based alloy. From the SEM image, it can be seen that the pore size of the corrosion layer is larger than that of the 304 stainless steel corrosion area. Figure 7a is a partial enlarged view. Unlike 304 stainless steel, 310S heat-resistant steel has distinct grains in all corrosion areas, smooth grain edges, and different grain sizes in different corrosion areas. The grain boundaries are filled with Sn alloy, and there are large pores between some grains that are micrometers in size.

Figure 7b shows a point scan of the 310S corrosion layer’s localized micro-area, with elemental composition results in Table 2. In the gray heat-resistant steel grain area, grain boundaries are severely corroded, and gaps are filled with Sn-Bi alloys. Zn is undetected at points F, G, H, and I. The large pores at I mainly contain Si, C, and O, likely formed during polishing when soft Sn alloy embedded diamond and SiO_2_. In region F, the presence of Sn indicates it penetrates steel grains more easily than Bi under high-temperature liquid metal corrosion. Region G is a Sn-rich area with Fe, Cr, and Ni detected, but no Bi or Zn. F has almost no Ni, while G has Ni. Since Ni is also undetected in the Bi-rich I region, it can be inferred that after 310S stainless steel corrosion, Ni combines with Sn from grain boundaries and the matrix. Ni promotes Sn and Bi segregation, causing phase separation of Sn-Bi alloys in the heat-resistant steel matrix.

Under high-temperature corrosion, a small amount of Ni element is distributed inside the grains of the corrosion layer, and a large amount of Ni element diffuses to the outside of the heat-resistant steel through the Sn alloy at the grain boundaries and integrates into the high-temperature Sn-based alloy liquid. Based on the distribution of the Ni element in stainless steel, the Ni element exists as a “harmful element” in the Fe matrix. In the high-temperature liquid Sn-Bi-Zn alloy, the Ni element has a greater tendency to dissolve, so the loss of Ni will cause a large number of pores in the matrix and damage the structure of the steel matrix.

**Figure 8:**
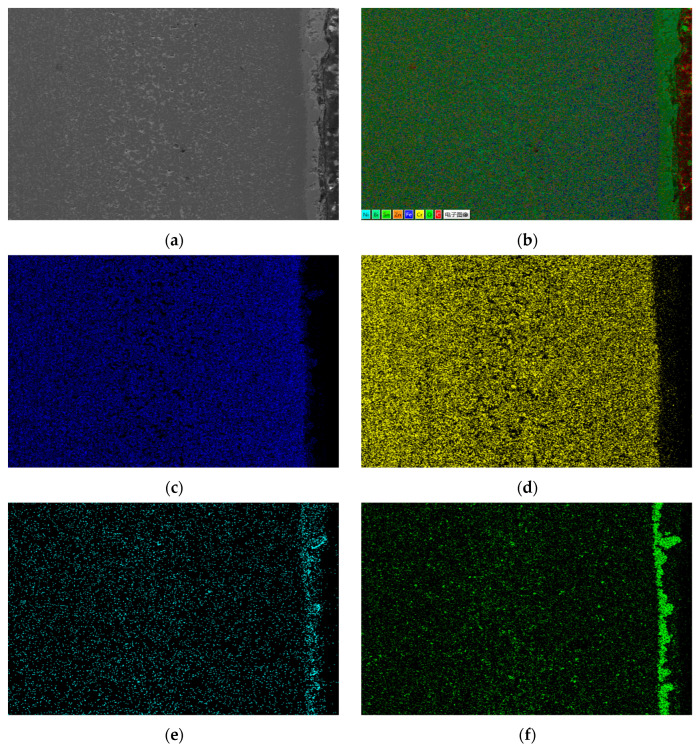

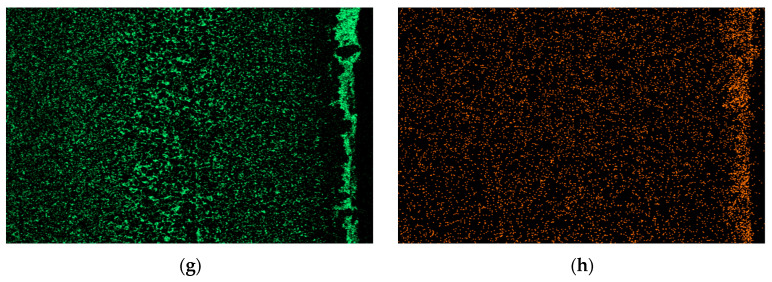
Element distribution of the cross-section of 310s heat-resistant steel after corrosion. (**a**) Surface scanning area; (**b**) EDS layered image; (**c**) Fe; (**d**) Cr; (**e**) Ni; (**f**) Sn; (**g**) Bi; (**h**) Zn.

Figure 8 presents the EDS surface scan and element distribution map of 301S heat-resistant steel’s corrosion section. Results show that Sn, Bi, and Zn diffuse significantly in the matrix, and Sn completely penetrates it. Like 304 stainless steel, Bi is less on the steel sheet surface, more in the middle of the corrosion layer, decreasing towards the surface and matrix interior. Zn has a low concentration and is evenly distributed in the matrix corrosion layer. Fe, Cr, and Ni in the matrix partly dissolve into the Sn alloy outside, with Ni having the highest solubility.

**Figure 9:**
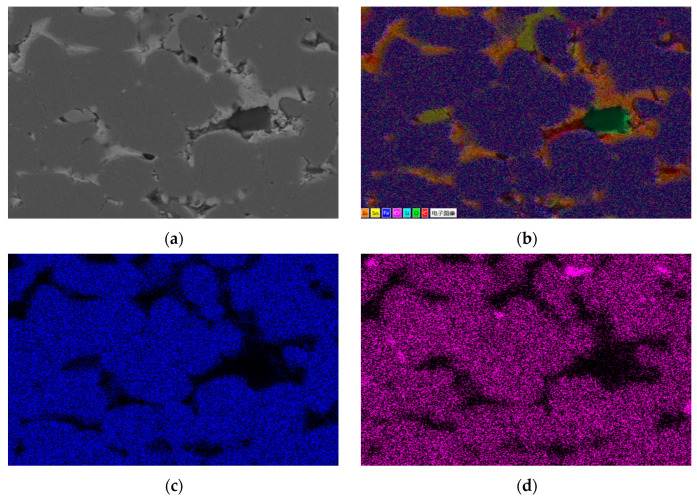

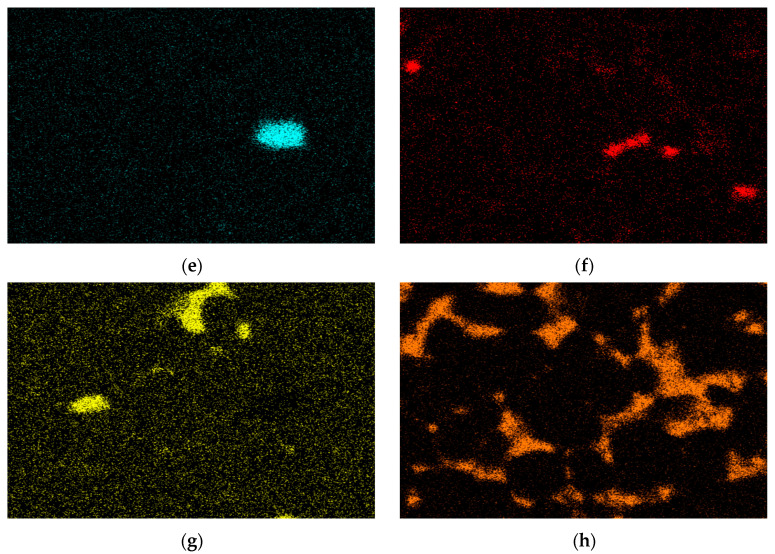
EDS surface scan results of micro elements in the corrosion layer of 310S heat-resistant steel. (**a**) Scanning area; (**b**) EDS layered image; (**c**) Fe; (**d**) Cr; (**e**) Ni; (**f**) C; (**g**) Sn; (**h**) Bi.

Figure 9 shows the EDS surface scan and element distribution map of the 310S corrosion layer micro-area. The EDS surface scan results show that the grain boundary corrosion is filled with elements such as Sn and Bi, which are prone to segregation after entering the stainless steel interior, The content of Zn element is relatively low, and the large pores are composed of Si and C, which are caused by diamond polishing. The results are consistent with the point scan analysis; The area occupied by the Bi element in the grain gaps is larger than that of the Sn element. Although Sn element is more likely to enter the interior of the matrix grains, overall, the Bi element is more likely to aggregate in the intergranular gaps inside the matrix.

#### 3.2.3. Element Distribution Results of 20 Carbon Steel After Corrosion

**Figure 10:**
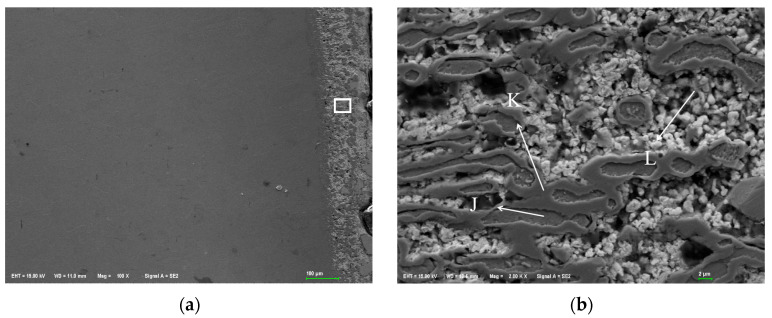
SEM morphology of corrosion layer on 20 carbon steel. (**a**) 100×; (**b**) 2000×.

Figure 10a shows the SEM corrosion morphology of a 20 carbon steel cross-section. At the same magnification, its corrosion layer is much thinner than that of stainless steel and heat-resistant steel, with a different surface morphology. Figure 10b, a partial enlarged view, reveals that for 20 carbon steel, corroded surface grains dissolve simultaneously with grain boundaries, not just starting from grain boundaries. Some matrix “falls off” to form “giant grooves”, distinct from the other two steels. In 304 stainless steel and 310S heat-resistant steel, corrosion areas create numerous pores at grain boundaries, with Sn, Bi, etc. filling grain gaps, and grains remain largely unchanged. At 600 °C insulation, the steel plate surface in contact with liquid metal becomes dense. Figure 10b also shows severe substrate damage, with outer corrosion area grains detaching from the internal substrate. Grains are filled with Sn alloy, but the filling alloy structure differs from that of stainless and heat-resistant steels. The filling metal in the 20 carbon steel’s corrosion area is particulate, while that of the other two steels is blocky. Further energy-spectrum analysis is needed for specific elemental composition.

Figure 10b shows the EDS point scan area of 20 carbon steel’s surface corrosion layer, with the results in Table 3. The corrosion area consists of three parts: Fe-dominated matrix, high-Cr area around the Fe matrix, and Bi-dominated alloy area. Small amounts of Sn and Zn infiltrate the Fe matrix, but only Sn in the corrosion layer diffuses, with no Sn in the matrix. Microscopically, the corroded area is surrounded by white granular tissue, with dark areas mainly of Fe. The surrounding Cr content is high, and C is enriched. The boundary between Cr and Bi is clear, showing repulsion; Bi-enriched areas have less Cr, and vice versa. Cr and C distributions are consistent. As Cr has a high affinity for C, this explains the accumulation of Cr on 20 carbon steel’s surface, which originally lacks Cr. In contrast, the Cr content in Bi-enriched areas is very low.

**Figure 11:**
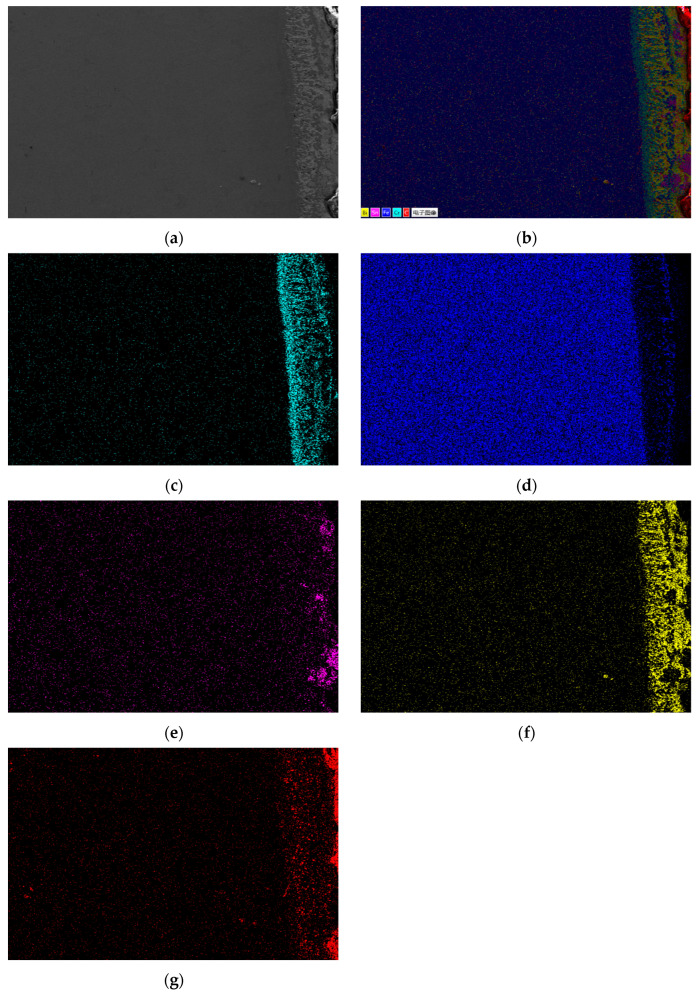
EDS surface scan elemental results of 20 carbon steel section. (**a**) Scan area; (**b**) EDS layered image; (**c**) Cr; (**d**) Fe; (**e**) Sn; (**f**) Bi; (**g**) C.

Figure 11 presents the EDS surface scan and element distribution map of the 20 carbon steel’s corroded section. Results indicate that Sn has limited diffusion in carbon steel. In stainless and heat-resistant steels, the strong affinity between Ni and Sn enables Sn to diffuse into the Fe matrix, causing Ni dissolution and void formation. Since 20 carbon steel contains little Ni, Sn diffusion is minimal, resulting in a much thinner corrosion layer compared to the other steels. Sn is mostly aggregated on the steel sheet surface with little presence inside. In contrast, Bi accumulates in the surface corrosion area and residual metal liquid. This verifies that during high-temperature corrosion by Sn-Bi-Zn alloy on Fe-based materials, Sn diffuses into the matrix, and Bi cracks the matrix along Sn’s diffusion path. Sn diffusion is positively related to Ni content, and Bi’s infiltration and corrosion ability depend on Sn diffusion.

**Figure 12:**
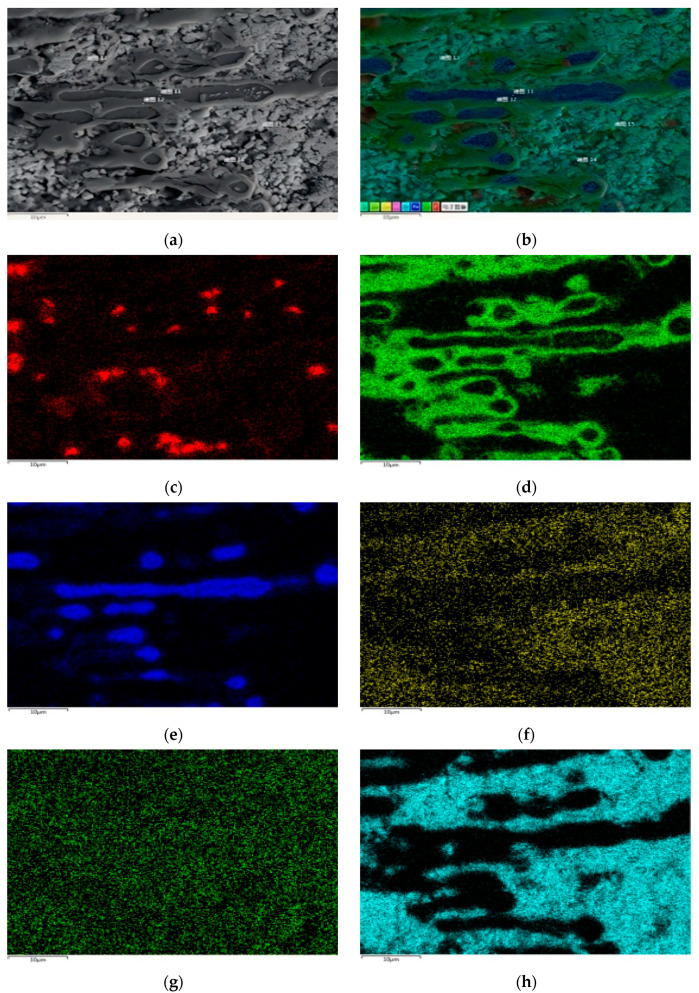
EDS surface scan results of localized corrosion layer inside 20 carbon steel. (**a**) Scanning area; (**b**) EDS layered image; (**c**) C; (**d**) Cr; (**e**) Fe; (**f**) Zn; (**g**) Sn; (**h**) Bi.

Figure 12 shows the partial surface scan and element distribution map of the corroded area of 20 carbon steel. There is a large amount of Cr element accumulation on the surface of 20 carbon steel. The reason is that the Cr element has a high affinity with the C element and is easy to form metal carbides with Fe and Cr. The heat-resistant steel, stainless steel, and 20 carbon steel used in the experiment are in the same corrosion container. During the corrosion process, the Cr element dissolved into the alloy liquid in the heat-resistant steel, and stainless steel had high atomic activity at high temperatures. At the same time, after corrosion occurs on the surface of the carbon steel, the C element continuously leaves the surface. Therefore, the concentration of C element on the surface of 20 carbon steel is much higher than that in the surrounding area, making it easy for the Cr element to accumulate on the surface of carbon steel.

### 3.3. Analysis of Diffusion Kinetics of Sn-Bi-Zn Alloy Components in Different Steels

The EDS energy spectrum analysis results indicate that the thickness of the corrosion layer is consistent with the diffusion distance of Sn, Bi, and Zn elements in the steel. Therefore, the diffusion distance of alloy elements in different steels can be directly obtained by calculating the corrosion thickness of the steel. This article uses the element Sn as a standard, calculates the diffusion coefficient of Sn element in steel according to Fick’s diffusion law, and quantitatively analyzes the diffusion behavior of Sn atoms in steel. The diffusion equation of dissimilar metals in steel is:

(1) $$C=C_{0}[1-erf(\beta )]$$

(2) $$\beta =\frac{x}{\sqrt{4Dt}}$$

where x is the diffusion distance, t is the diffusion time, C_0_ is the initial concentration, C is the concentration at the diffusion distance of x, and D is the diffusion coefficient.

There is a linear relationship between C (x, t) and x/$\sqrt{4Dt}$. The diffusion coefficient D was calculated using Fick’s second law. For a fixed concentration C at the corrosion front, the relationship x^2^ = K(C)·t was derived, where K(C) = 4Dβ^2^. Here, β is determined by the error function solution to Fick’s law for the selected C. By plotting x^2^ versus t, the slope K(C) was obtained, enabling the calculation of D. This method ensures K(C) remains consistent for the defined concentration threshold, validating its use as a concentration-dependent constant in our analysis.

**Figure 13:**
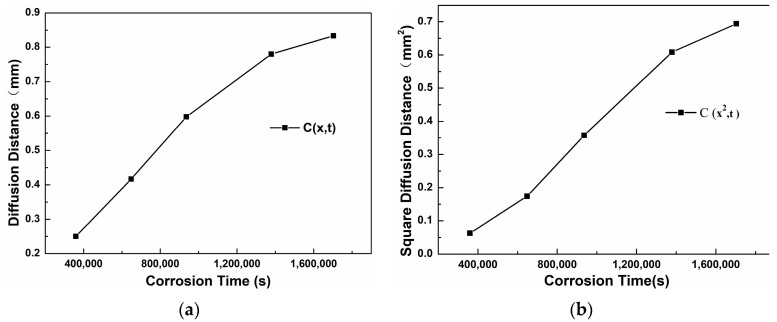
Corrosion layer curve of 304 stainless steel. (**a**) x − t; (**b**) x^2^ − t.

**Figure 14:**
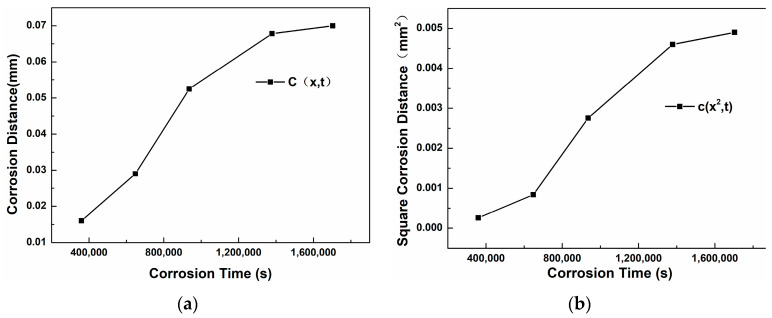
Corrosion layer curve of 20 carbon steel. (**a**) x − t; (**b**) x^2^ − t.

The thickness of the corrosion layer of the steel was obtained by an optical microscope. The diffusion coefficient D was calculated using the relationship x^2^ = K(C)⋅t, where x is the corrosion layer thickness and K(C) is a proportionality constant. This approach aligns with the mean square displacement framework (<x^2^> = 2Dt) but incorporates additional factors specific to the steel microstructure and Sn-Bi-Zn interaction. By plotting x^2^ versus t (Figure 13 and Figure 14), the slope K(C) was obtained. Using the error function solution to Fick’s law, K(C) was expressed as 4Dβ^2^, where β is determined by the concentration threshold at the corrosion front. The derived D values reflect the combined effects of lattice and grain boundary diffusion, consistent with the observed corrosion severity across steel types. The average value of the thicknesses of the corrosion layers at ten different positions was taken, and the specific results are shown in Table 4.

Figure 13a shows the trend curve of the thickness of the corrosion layer of 304 stainless steel changing with the corrosion time. It can be seen that as the corrosion time increases, the slope of the curve becomes smaller, indicating that the diffusion rate decreases with the increase of time. Figure 13b is the curve of x^2^ changing with t. Through linear fitting, the equation x^2^ = 4.97 × 10^−7^ t is obtained, that is, K(C) = 4.97 × 10^−7^. The assumption erf(β) ≈ 1 was based on a threshold concentration C = 0.01C_0_, corresponding to β ≈ 1.82. While erf(1.82) = 0.99, this approximation simplifies calculations without significantly affecting D, as confirmed by refined analysis. Further calculation gives the diffusion coefficient D_304_ = K(C)/4β^2^ = 3.7 × 10^−8^ mm^2^/s.

It can be seen from Table 5 that 310S heat-resistant steel completed corrosion after 180 h, with a corrosion distance of 1.500mm, K(C) = 3.2639 × 10^−6^. At 600 °C corrosion, the diffusion coefficient D310S of alloy elements inside the 310S matrix is 2.51 × 10^−7^ mm^2^/s, so the high-temperature corrosion resistance of 310S heat-resistant steel is extremely poor, far inferior to stainless steel.

Table 6 shows the thickness of the corrosion layer of 20 carbon steel after different corrosion times. Figure 14a is the curve of the diffusion distance changing with the corrosion time. It can be seen that as the time increases, the slope of the curve first becomes larger from a smaller value and then decreases and tends to be stable. Figure 14b is the curve of the square of the diffusion distance changing with time. Through linear fitting, the equation x^2^ = 3.81 × 10^−9^ t is obtained, that is, K(C) = 3.81 × 10^−9^. Further calculation gives the diffusion coefficient D_20C_= K(C)/4β^2^ = 2.87 × 10^−10^ mm^2^/s.

Through comprehensive comparison, the calculated diffusion coefficients are D_20C_ < D_304_ < D_310S_. The diffusion rate of Sn element is the highest in 310S heat-resistant steel and the lowest in 20 carbon steel. EDS energy spectrum analysis shows that in the corrosion areas of 310S and 304, a large amount of Ni element is lost. In stainless steel and heat-resistant steel, the Sn element infiltrates through grain boundaries, and there is a concentration gradient between the Ni element and grain boundaries, which promotes the continuous precipitation of the Ni element from between grains, migration to grain boundaries, and finally loss to the liquid metal. The results show that in stainless steel and heat-resistant steel, the Ni element exists in the form of solid solution and intermetallic compounds in Fe crystals, and Ni and Fe elements maintain equilibrium with each other. When stainless steel and heat-resistant steel are in liquid Sn alloy, the Sn element diffuses into Fe crystals due to the concentration gradient, breaking this equilibrium. This causes the continuous precipitation of Ni element solid solution in Fe crystals. Therefore, the diffusion rate of Sn element in Ni-containing stainless steel and heat-resistant steel is much higher than that in 20 carbon steel without Ni element.

### 3.4. Analysis of the Dissolution Process of Steel in High-Temperature Molten Metal

**Figure 15:**
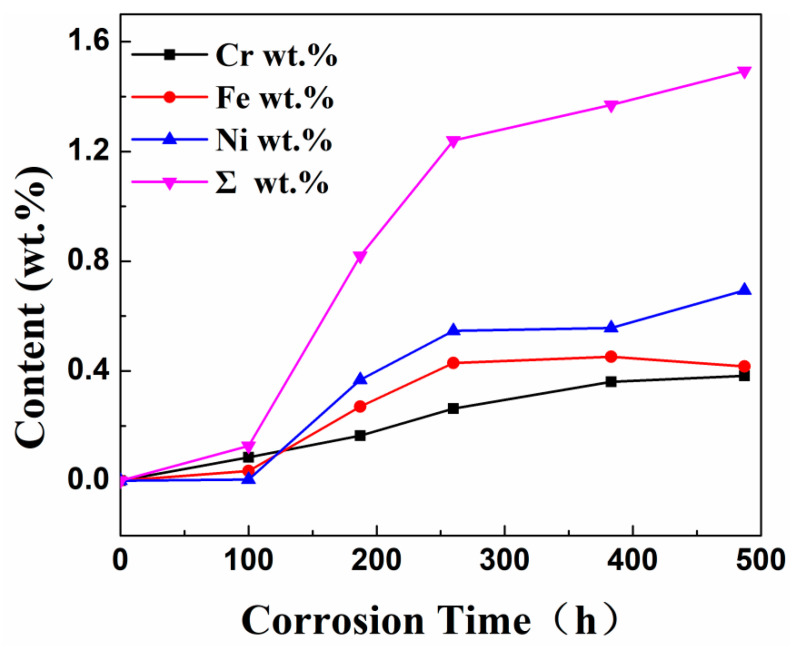
The curve of the mass fraction of Fe, Cr, and Ni elements dissolved into the Sn-Bi-Zn alloy over time.

Table 7 shows the XRF fluorescence elemental analysis results of the Sn-Bi-Zn alloy after corrosion. Fe, Cr, and Ni elements are present in the corroded alloy solution. Figure 15 shows that as the corrosion time increases, the content of Fe, Cr, and Ni elements gradually increases, indicating that the steel sheet is exposed to the liquid high-temperature Sn-Bi-Zn alloy. While Sn, Bi, and Zn diffuse into the steel interior, the steel components also dissolve into the alloy interior.

**Figure 16:**
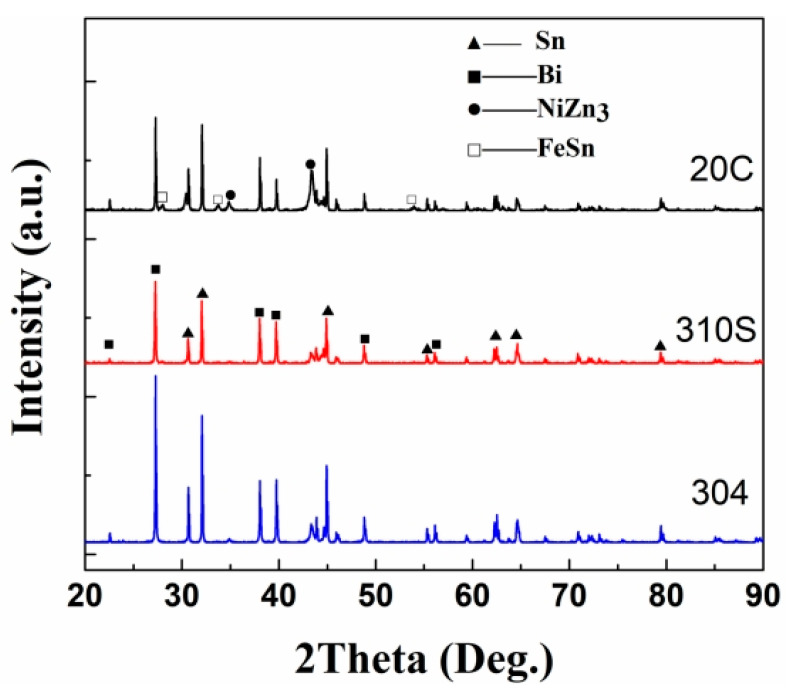
XRD diffraction patterns of corroded surfaces of different steels.

The surface phase composition of the material was analyzed using an X-ray diffractometer, and the results are shown in Figure 16. The XRD analysis results show that in the XRD pattern of 304 stainless steel, in addition to the diffraction peaks of Sn and Bi, the diffraction peaks of the intermetallic compounds NiZn_3_ and FeSn also appear. Considering the Ni-Zn alloy phase diagram [29], the solidus temperature of NiZn_3_ is 419.58 °C, and it forms in the Zn-rich region. In the energy-dispersive X-ray spectroscopy (EDS) surface scanning and point scanning element results of 304 stainless steel, almost no Ni element was detected in the corrosion layer, while the Ni element was concentrated in the surface area. It can be judged that the loss of Ni element is the result of the combined effects of Sn and Zn elements. Sn and Zn elements diffuse into the grain interior, changing the equilibrium relationship of the Fe(Ni) solid solution and simultaneously promoting the loss of Ni in the steel. Comparing the diffraction patterns of the three steels, the diffraction peak of NiZn_3_ exists in all of them. However, compared with 304 stainless steel, the diffraction peaks of NiZn_3_ in 310S heat-resistant steel and 20 carbon steel are very weak, indicating a relatively low content of this substance. The diffraction peak of the phase FeSn appears in 304 stainless steel, but it does not appear in 310S heat-resistant steel and 20 carbon steel. By referring to relevant literature, the Cr content in 310S reaches 18%, and the C content in 20 carbon steel is high. The affinity of Cr and C for Fe is much greater than that of Sn for Fe. At the same time, the Ni content in 304 is relatively high. The loss of Ni promotes the combination of Fe and Sn, resulting in the existence of the FeSn phase in 304, while it is absent in 310S and 20C. In the XRD pattern of the 304 stainless steel surface, the diffraction peaks of NiZn_3_ and FeSn appear. Based on the analysis of the Fe-Sn and Ni-Zn phase diagrams reported in ASM Handbook Volume 3 [29], Fe and Ni elements form intermetallic compounds (e.g., FeSn, NiZn_3_) on the surface during corrosion. These phases gradually dissolve into the liquid alloy due to chemical potential gradients. However, currently only the NiZn_3_ and FeSn phases are detected, indicating that although there are phase diagrams, the combination tendencies between different elements are different.

The diffusion of metal elements essentially belongs to the chemical potential gradient. In the corrosion test of this paper, the dissolution of Ni and Fe elements into the liquid metal must reduce the free energy. As shown in Figure 16, the existence of intermetallic compounds NiZn_3_ and FeSn is detected on the steel surface. Therefore, it can be determined that in addition to the concentration gradient driving force, there is also a chemical gradient driving force for the diffusion of Ni and Fe. In a closed system, the system always tends to move from a state of high free energy to a state of low free energy. Therefore, when the steel is exposed to the liquid alloy, affected by the chemical potential gradient, Ni and Sn are continuously combined with Zn and Sn to form intermetallic compounds and are continuously dissolved into the heat transfer alloy.

## 4. Discussion

The high-temperature corrosion behavior of Sn-Bi-Zn alloy observed in this study aligns with and expands upon findings from previous investigations on liquid metal corrosion. Below, we compare our results with those reported in the literature, highlighting key similarities, discrepancies, and novel insights.

Our experiments revealed that 310S heat-resistant steel (high Ni content) exhibited the most severe corrosion (D310S = 2.51 × 10^−7^ mm^2^/s), while 20 carbon steel (no Ni) showed the lowest corrosion rate (D20C = 2.87 × 10^−10^ mm^2^/s). This trend is consistent with studies by Emmerich et al. [24], who reported accelerated Ni dissolution in austenitic steels exposed to liquid Sn at 500 °C. Similarly, Kang et al. [22,23] demonstrated that Ni dissolution in liquid Sn follows a linear kinetic model, with Ni loss increasing proportionally to temperature and exposure time. Our findings extend these observations by linking Ni depletion to pore formation and grain boundary weakening, a mechanism less emphasized in earlier works.

The significant diffusion of Sn into stainless steel (D304 = 3.7 × 10^−8^ mm^2^/s) and its accumulation at grain boundaries corroborate findings by Kania [27], who observed Sn-induced intergranular cracking in Zn-Bi-Sn baths. However, Kania’s study focused on lower temperatures (450 °C), whereas our work demonstrates that at 600 °C, Sn diffusion is further amplified by Ni depletion, a factor not previously quantified.

Unlike Sn, Bi showed limited diffusion into the steel matrix but aggregated at grain boundaries (Figure 5g and Figure 8g). This aligns with Li et al. [28], who noted that Bi segregates to Sn diffusion paths in Mg-Zn-Ca alloys, exacerbating matrix cracking. Our study adds mechanistic clarity by showing that Bi accumulation destabilizes the Fe-Ni-Cr solid solution, accelerating Ni loss (Table 1 and Table 2).

This work bridges gaps between existing liquid metal corrosion models and multi-component alloy interactions. By integrating experimental data with Fickian kinetics and phase diagram analysis, we provide a framework for predicting material compatibility in next-generation heat transfer systems. Our findings underscore the necessity of alloy-specific corrosion assessments, particularly for applications involving ternary or quaternary liquid metals.

## 5. Conclusions

This study systematically investigated the high-temperature corrosion behavior of Sn-50Bi-2Zn (wt.%) alloy against three structural steels (304 stainless steel, 310S heat-resistant steel, and 20 carbon steel) at 600 °C. Key findings, supported by quantitative measurements and comparative analysis, are summarized as follows:

(1) 310S heat-resistant steel exhibited the most severe corrosion, with a corrosion layer thickness of 1.50 mm after 180 h—81% thicker than 304 stainless steel (0.83 mm after 473 h) and 21.4× greater than 20 carbon steel (0.07 mm after 473 h). The diffusion coefficient of Sn in 310S (D310S = 2.51 × 10^−7^ mm^2^/s) was 6.7× higher than in 304 stainless steel (D304 = 3.7 × 10^−8^ mm^2^/s) and 875× higher than in 20 carbon steel (D20C = 2.87 × 10^−10^ mm^2^/s).
(2) Ni loss dominated in stainless and heat-resistant steels, with 304SS losing 7.72 wt.% Ni at the corrosion front (Table 1) and 310S dissolving 0.694 wt.% Ni into the alloy after 480 h (Table 7). This Ni dissolution directly correlated with pore formation, occupying ~15% of the corrosion layer volume in 310S (Figure 8b); 20 carbon steel, lacking Ni, showed minimal Sn diffusion (<0.05 mm penetration depth) and retained 97% of its original Fe content in the substrate (Table 3).
(3) The XRF fluorescence analysis confirmed the dissolution of Fe, Cr, and Ni elements from the steel substrates into the molten Sn-Bi-Zn alloy, with Ni exhibiting the highest solubility (up to 0.694 wt.% after 480 h). Intermetallic compounds, including NiZn_3_ and FeSn, were identified on the surface of 304 stainless steel through XRD, while these phases were absent in 310S heat-resistant steel and 20 carbon steel. This distinct phase formation in 304 stainless steel highlights its propensity to dissolve into the liquid metal via intermetallic reactions, driven by the strong chemical affinity between Sn/Ni and Sn/Fe. In contrast, the lack of NiZn_3_ peaks in 310S and 20 carbon steel correlates with their lower Ni content and Cr-dominated surface interactions, respectively. These results underscore the critical role of Ni content in accelerating corrosion through intermetallic compound formation, providing a mechanistic basis for material degradation in high-temperature liquid metal environments.

## Figures and Tables

**Table 1:** EDS point scan elemental composition results of corrosion micro-areas on the surface of 304 stainless steel.

Point/Element/wt.%	Fe	Cr	Ni	Sn	Bi	Zn	C	Si
A	68.77	17.80	7.72	-	-	-	5.12	0.60
B	77.59	13.45	-	3.33	-	1.30	4.33	-
C	73.52	13.55	-	2.92	-	1.34	7.51	-
D	60.75	11.36	-	15.01	0.71	1.49	7.72	-
E	45.49	9.09	-	2.43	36.10	0.85	5.58	0.45

**Table 2:** EDS point scan elemental composition results of corrosion micro-areas on the surface of 310S heat-resistant steel.

Point/Element/wt.%	Fe	Cr	Ni	Sn	Bi	Zn	C	Si	O
F	71.98	18.28	-	3.25	-	-	5.72	0.77	-
G	23.63	3.82	3.39	62.15	-	-	7.01	-	-
H	1.70	1.13	-	0.62	7.34	-	11.36	33.59	44.26
I	9.66	2.96	-	0.99	78.08	-	8.30	-	-

**Table 3:** EDS point scan elemental composition results of corrosion micro-areas on the surface of 20 carbon steel.

Point/Element/wt.%	Fe	Cr	Ni	Sn	Bi	Zn	C	Si
J	80.16	7.71	0.13	2.47	0.38	1.83	6.23	0.38
K	15.16	69.23	-	0.33	-	0.31	14.73	0.23
L	1.49	1.05	0.12	0.20	86.10	0.39	7.13	-

**Table 4:** Corrosion layer thickness of 304 stainless steel at different times.

Time/Thickness (mm)	1	2	3	4	5	6	7	8	9	10	Average Value
100 h	0.22	0.21	0.18	0.21	0.32	0.29	0.40	0.30	0.20	0.17	0.25
180 h	0.39	0.38	0.41	0.41	0.42	0.43	0.44	0.43	0.41	0.45	0.42
260 h	0.65	0.62	0.66	0.67	0.63	0.53	0.67	0.55	0.49	0.51	0.60
383 h	0.78	0.77	0.76	0.77	0.81	0.82	0.82	0.77	0.76	0.78	0.78
473 h	0.84	0.84	0.85	0.79	0.77	0.86	0.86	0.87	0.83	0.82	0.83

**Table 5:** Corrosion layer thickness of 310S heat-resistant steel at different times.

Time/Thickness (mm)	1	2	3	4	5	6	7	8	9	10	Average Value
100h	0.56	0.53	0.42	0.58	0.42	0.55	0.60	0.62	0.65	0.59	0.55
180h	1.50	1.50	1.50	1.50	1.50	1.50	1.50	1.50	1.50	1.50	1.50

**Table 6:** Corrosion layer thickness of 20 carbon steel at different times.

Time/Thickness (mm)	1	2	3	4	5	6	7	8	9	10	Average Value
100 h	0.0250	0.0225	0.0075	0.0275	0.0075	0.0150	0.0100	0.0200	0.0175	0.0075	0.0160
180 h	0.0150	0.0175	0.0175	0.0150	0.0225	0.0425	0.0400	0.0475	0.0350	0.0375	0.0290
260 h	0.0625	0.0625	0.0600	0.0400	0.0650	0.0400	0.0575	0.0350	0.0525	0.0500	0.0525
383 h	0.7800	0.7700	0.7600	0.7700	0.8100	0.8200	0.8200	0.7700	0.7600	0.7800	0.0678
473 h	0.0700	0.0750	0.0750	0.0725	0.0850	0.0775	0.0650	0.0850	0.0775	0.0875	0.0700

**Table 7:** Analysis results of Fe, Cr, and Ni content in the Sn-Bi-Zn alloy after corrosion.

Element/Time/Content wt.%	100 h	180 h	260 h	380 h	480 h
Fe	0.086	0.164	0.263	0.361	0.382
Cr	0.036	0.270	0.429	0.452	0.417
Ni	0.005	0.368	0.546	0.557	0.694
Percentage	0.127	0.82	1.24	1.370	1.493

## Data Availability

The original contributions presented in this study are included in the article. Further inquiries can be directed to the corresponding author.
